# Supplementary figures and images for: MeV-Stealth: A CD46-specific oncolytic measles virus resistant to neutralization by measles-immune human serum
Source: PLoS Pathog. 2021 Feb 3;17(2):e1009283. doi: 10.1371/journal.ppat.1009283 (PMC7886131; doi:10.1371/journal.ppat.1009283)

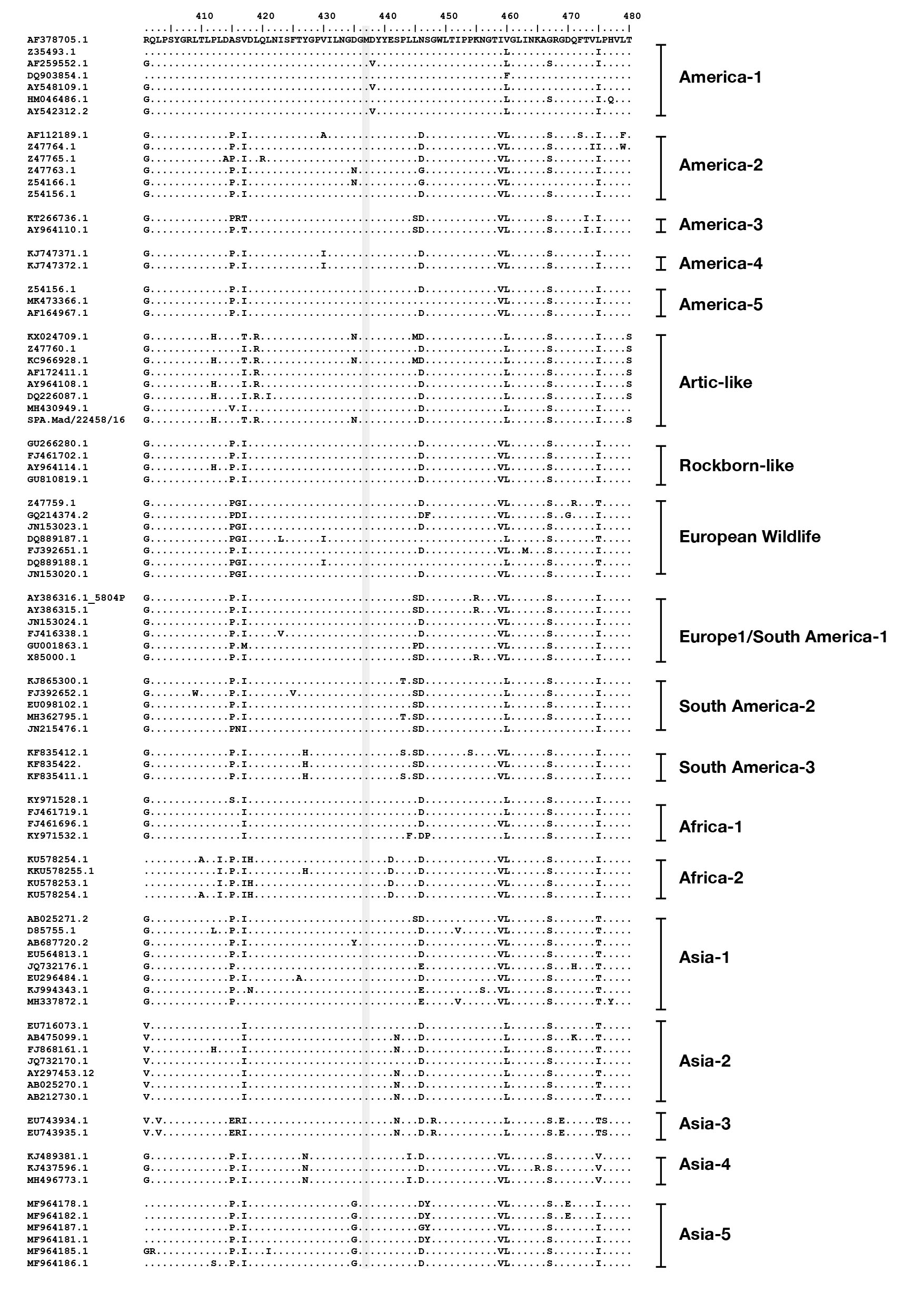

Supplement: S1 Fig — Sequence alignment was performed with CDV-H sequences retrieved from GenBank, including the CDV-H sequence determined here for the SPA.Madrid/22458/16 isolate. The accession numbers are indicated. (TIF) [file ppat.1009283.s001.tif]

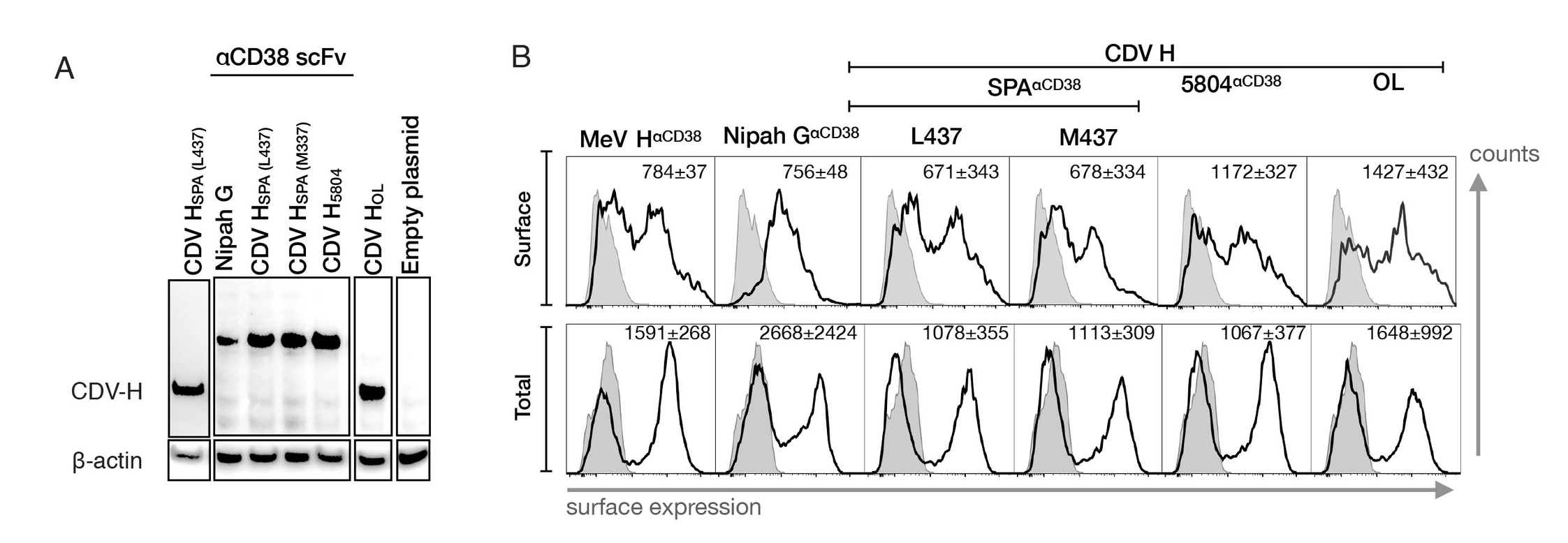

Supplement: S2 Fig — (A) Western blot analysis of HEK293T cells transfected with the indicated proteins fused to an anti-CD38 scFv or not. Proteins were blotted with an anti-HIS antibody or an anti-β-actin antibody (loading control). (B) Protein expression of the attachment proteins and mutants on HEK293T cells fixed with or without permeabilization analyzed by flow cytometry. Histograms are from one representative experiment out of two biological replicates. Geometric mean intensity ± SD from two biological replicates is shown at the upper right corner of each histogram. Filled curves denote cells transfected with empty plasmids. (TIF) [file ppat.1009283.s002.tif]

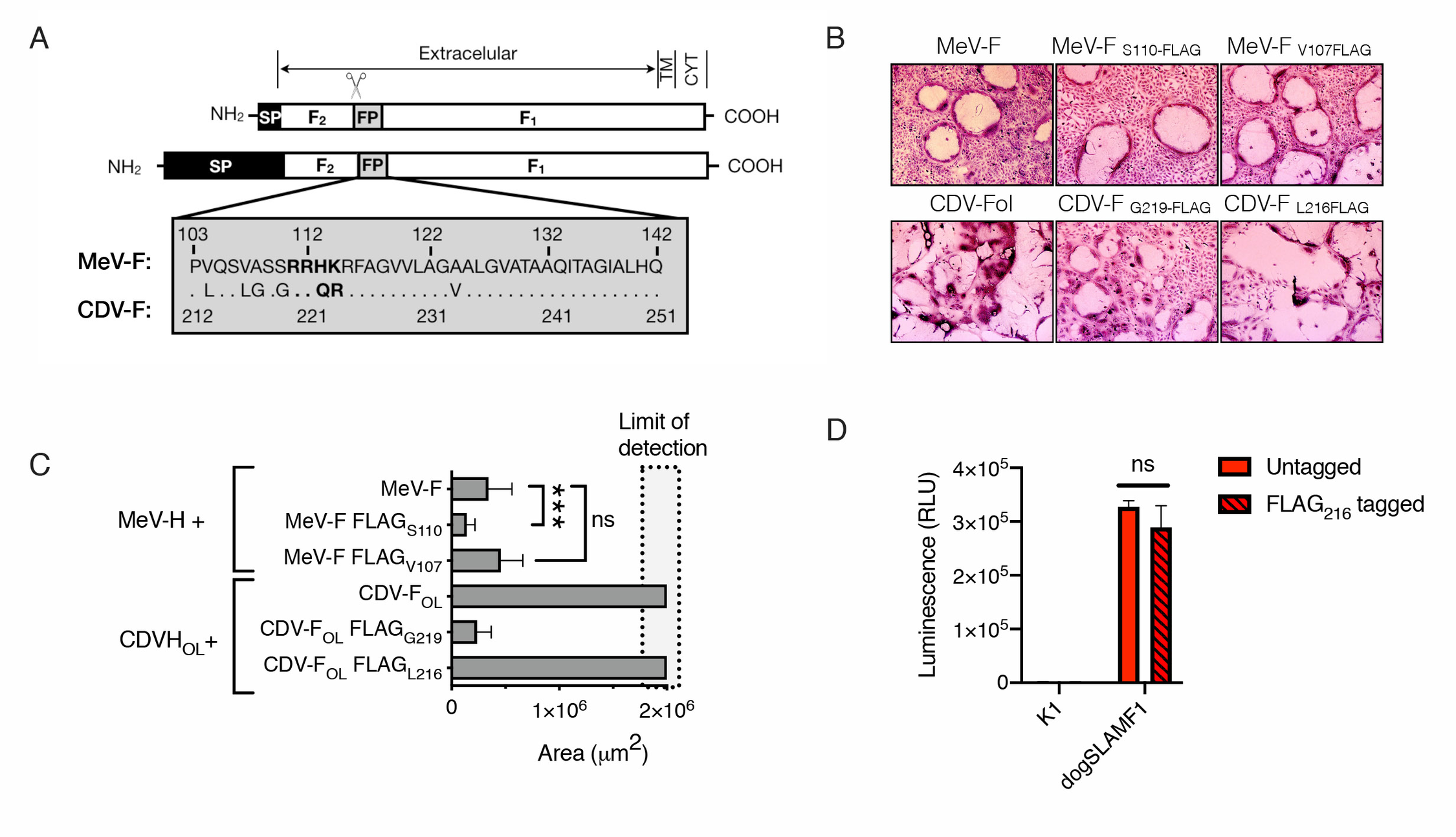

Supplement: S3 Fig — (A) Schematic drawings of uncleaved MeV-F and CDV-F. The NH2 and COOH termini, signal peptide (SP), fusion peptide (FP), and transmembrane (TM) and cytoplasmic regions are indicated. The sequence surrounding the cleavage site (in bold) and that of the fusion peptide are shown. The numbering considers the homotypic signal peptides. (B) Syncytia formation in Vero cells after cotransfection of homologous H and F expression plasmids with FLAG insertions at different positions. Cells were stained at 16 hours posttransfection, and microphotographs were acquired for quantification. (C) Quantification of syncytia formation. The data are shown as the mean ± SD (n = 20). Significance was determined using one-way ANOVA with Holm-Sidak’s multiple comparison test (ns, not significant; ***, p≤0.001). (D) Dual-split protein fusion assay for the cotransfection of CDV-H/F SPA with or without a FLAG-tag insertion at aa 216. The luciferase signal was measured at 8 hours. The experiment was performed in technical duplicates. (TIF) [file ppat.1009283.s003.tif]

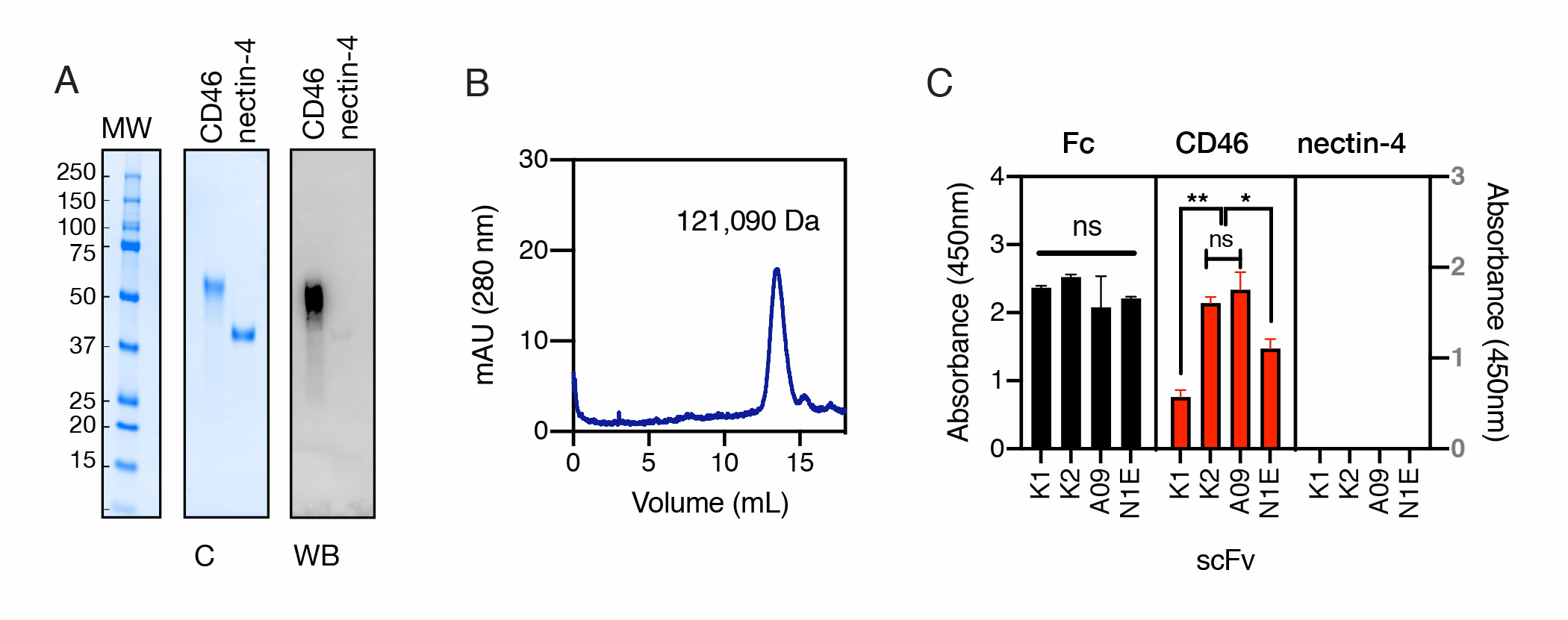

Supplement: S4 Fig — (A) SDS-PAGE analysis of target proteins. MW: molecular weight ladder, C: Coomassie staining; WB: western blot analysis using an anti-CD46 antibody. (B) Size exclusion chromatography trace for the CD46 used in the experiments. The estimated MW from a calibration curve is indicated. (C) Binding of scFv-Fc tagged fusion proteins to CD46 or nectin-4 as determined by ELISA. Detection was performed with the Fc portion used as a control for the amount of protein. Experiments were performed in technical duplicates. The data are shown as the mean ± SD, n = 2). Significance was determined using one-way ANOVA with Holm-Sidak’s multiple comparison test. *, p<0.05; **, p<0.005. (TIF) [file ppat.1009283.s004.tif]

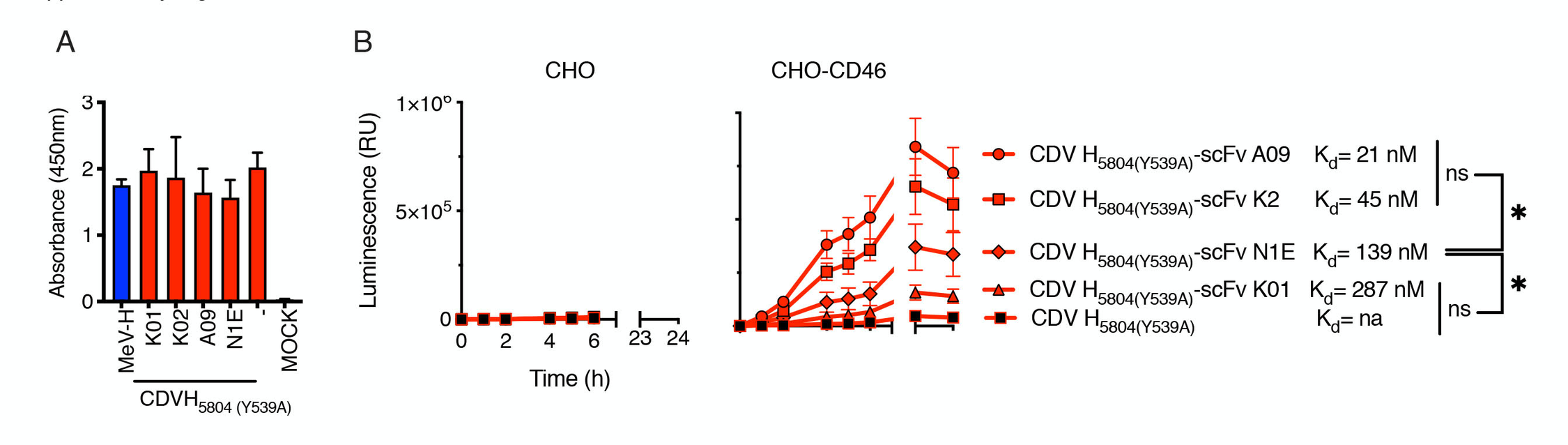

Supplement: S5 Fig — Binding affinity of the scFv displayed onto the CDV-H/F complex drives enhanced cell-cell fusion. (A) Cellular enzyme-linked immunosorbent assay (CELISA) for the amount of cellular protein used in the quantitative fusion assay on Fig 3C. A CELISA was performed on CHO cells transfected with the indicated attachment protein using an anti-6× HIS-tag monoclonal antibody (n = 5). (B) Quantitative fusion assay for the CD46-retargeted CDV-H/F complex using affinity tuned scFvs (same data as presented in Fig 3C). Y539A indicates the substitution in CDV-H to ablate the natural tropism for nectin-4. (TIF) [file ppat.1009283.s005.tif]

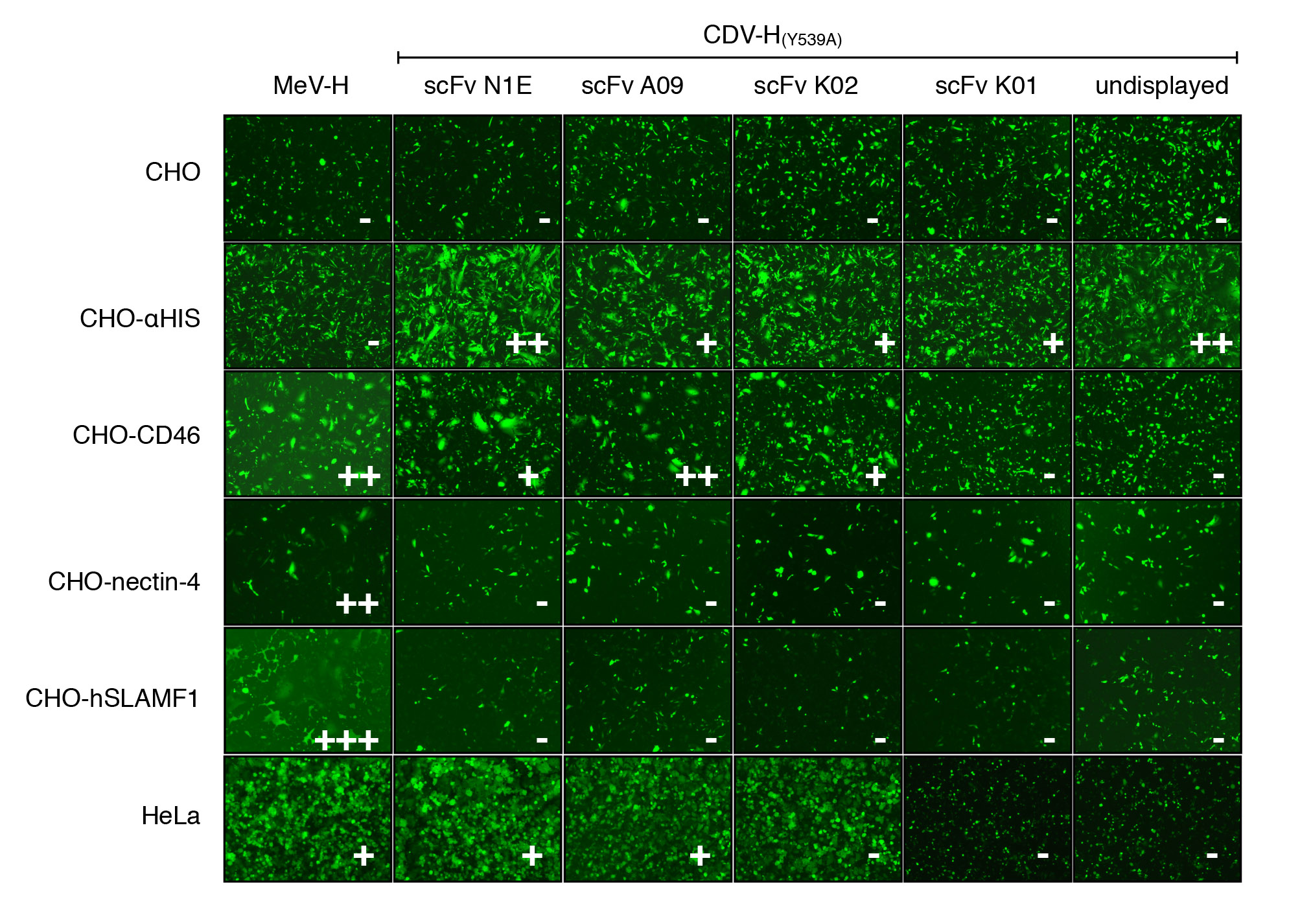

Supplement: S6 Fig — Cells were cotransfected with MeV-F and MeV-H or CDV-F and CDV-H retargeted variants with a CD46-specific scFv. For visualization purposes, an expression plasmid encoding eGFP was cotransfected, and eGFP autofluorescence was visualized at 24 hours posttransfection. Y539A indicates the substitution in CDV-H to ablate the natural tropism for nectin-4. “+” and “–” symbols were used for semiquantification (same as presented in Fig 2A). “Undisplay” indicates no scFv. (TIF) [file ppat.1009283.s006.tif]

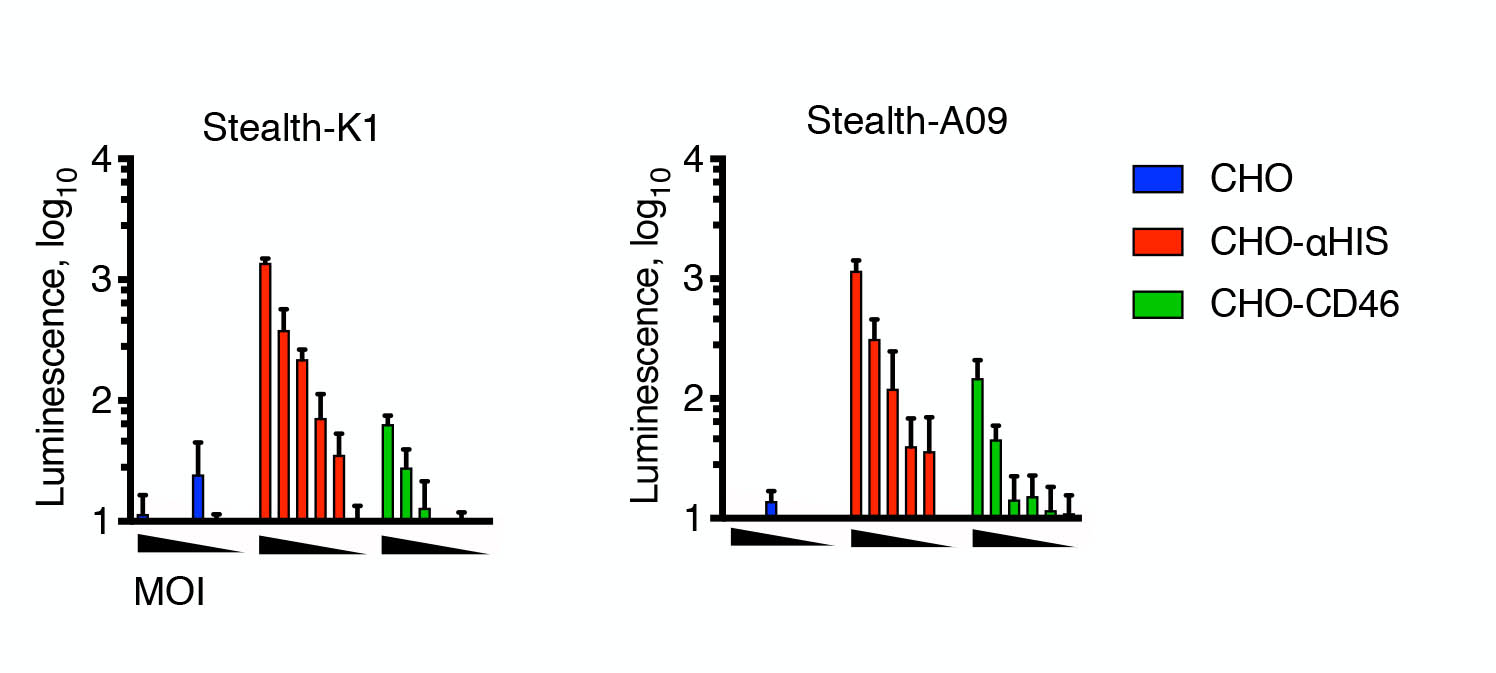

Supplement: S7 Fig — Increased binding affinity to CD46 enhances CD46-specific virus entry. Fluc-expressing Stealth viruses were used to infect the indicated cells at decreasing MOI. Luciferase expression was measured 48h postinfection. n = 2 for all except CHO-CD46 and Stealth-A09 (n = 3). (TIF) [file ppat.1009283.s007.tif]

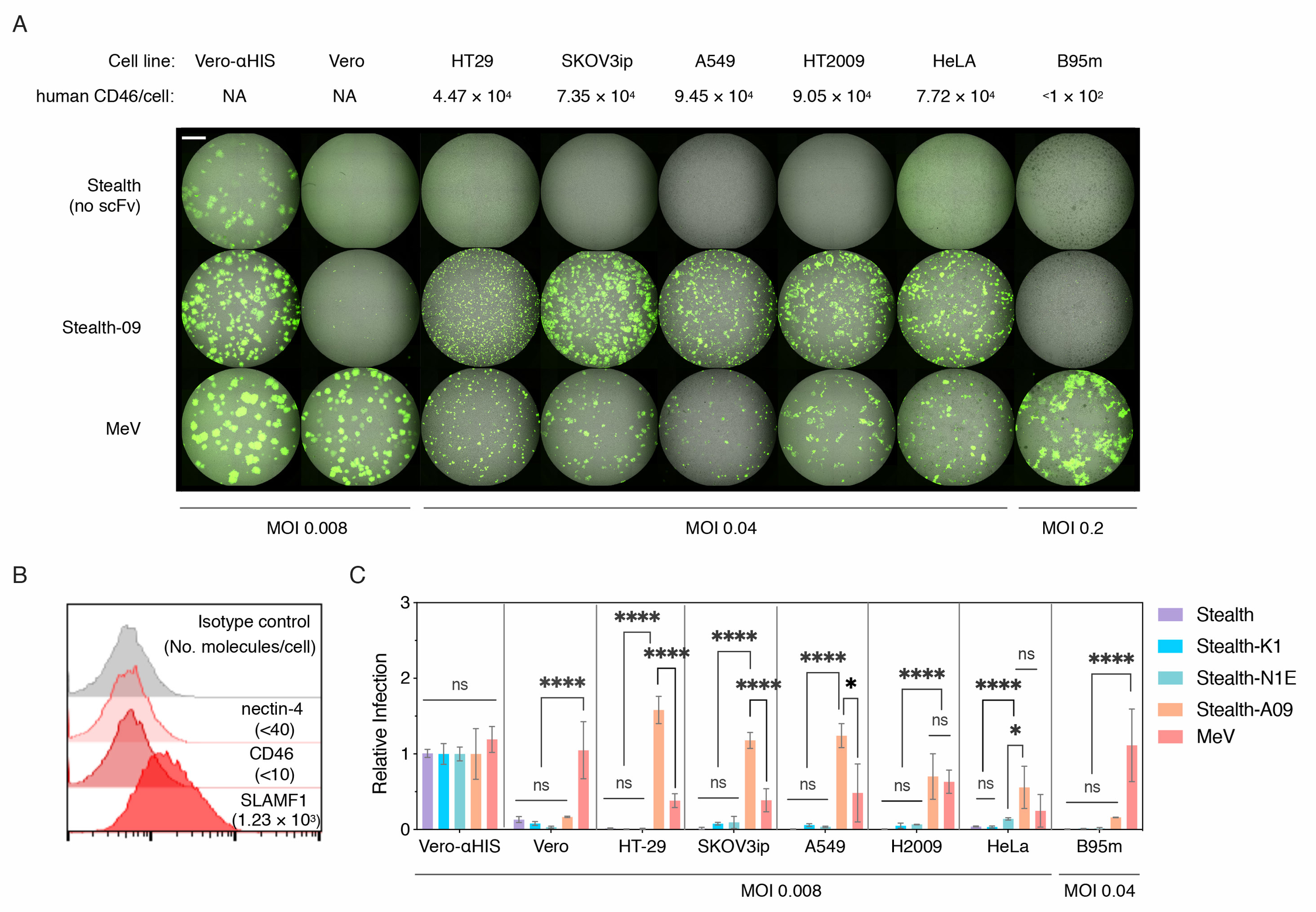

Supplement: S8 Fig — (A) A panel of immortalized cell lines was infected with parental MeV, MeV-Stealth and MeV-Stealth displaying CD46-specific scFv-A09 (Stealth-A09). Representative overlay of bright-field and fluorescence images were taken 48 hours post-infection. The number of CD46 molecules/cell is indicated for each cell line. NA, not applicable (i.e., the anti-human CD46 antibody does not cross-react with African green monkey kidney [Vero] cells). Different MOI was used for visualization purposes. (B) Flow-cytometric analysis of the cell surface expression of MeV-receptors on B95m cells after incubation with PE-specific antibodies. The determined number of molecules per cell is indicated in brackets. (C) Quantification of infection of cell lines with MeV and MeV-Stealth. The data are shown as the mean ± SD (n = 3). All data were analyzed 48 hours post-infection and are normalized to the respective infectivity of the indicated virus in Vero-αHIS. Significance was determined using two-way ANOVA with Turkey’s multiple comparison test. Ns, non-significant; p>0.05; *, p<0.0366; ****, p<0.0001. Scale bar, 1 mm. (TIF) [file ppat.1009283.s008.tif]

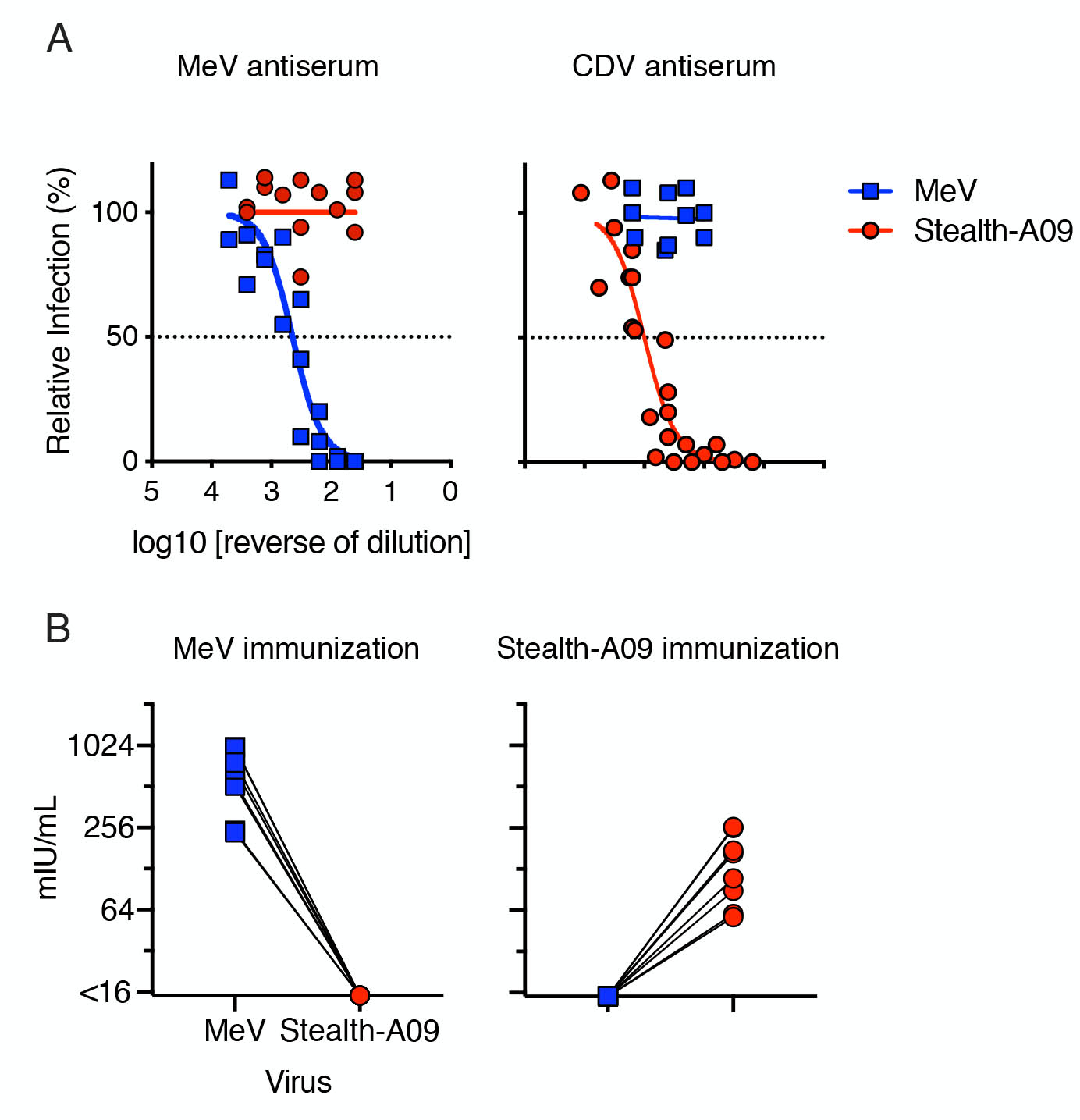

Supplement: S9 Fig — (A) Virus neutralization assay for MeV and Stealth. Human AB pooled serum (left panel) or ferret anti-CDV serum (right panel) was used. Relative infection refers to the amount of infection in the presence of serum compared with that in the absence of serum. Values were calculated from two or three biological replicates performed in technical quadruplicates. (B) Antisera from infected Ifnartm-CD46Ge mice was also used to determine the cross-neutralization between the viruses, n = 8 (note that some data points overlap). ND50 titers were converted to mIU/mL based on the ND50 obtained for MeV when assessed with the 3rd WHO International serum standard (3 IU/mL). (TIF) [file ppat.1009283.s009.tif]
