# Supplementary material for: MeV-Stealth: A CD46-specific oncolytic measles virus resistant to neutralization by measles-immune human serum
Source: PLoS Pathog. 2021 Feb 3;17(2):e1009283. doi: 10.1371/journal.ppat.1009283 (PMC7886131; doi:10.1371/journal.ppat.1009283)
Supplement: Attachment — Submitted filename: Response to reviewers.docx [file ppat.1009283.s011.docx]

Reviewer's Responses to Questions

**Part I - Summary**
Please use this section to discuss strengths/weaknesses of study, novelty/significance, general execution and scholarship.

Reviewer #1: The Russell laboratory, one of the most authoritative in the OV field, pioneered the field of MeV retargeting to cancer-specific receptors (ref 26). Here, Muñoz-Alía et al report on a strategy for MeV retargeting to CD46 which also confers evasion from human anti-MeV antibodies. The retargeting principle is based on swapping of MeV glycoproteins with the CDV homologs, and retargeting to CD46 by scFv insertion.

The initial experiments were carried out to select a Canine Distemper Virus (CDV) strain capable of using CD-46 and of high fusogenic activity towards CD46+ cells, as well as towards cells positive for nectin4 or SLAMF1.

The assay for selection of the best CDV strain and of the best combination of N and F was also based on a cell-cell fusion assay. Based on the same assay, the Authors then selected a scFv to CD46, and, furthermore, introduced single amino acid substitutions for detargeting from nectin4 and from SLAMF1.

The Authors then constructed the MeV-Stealth virus. When tested against a panel of CHO derivatives Stealth is retargeted to Cd46, detergeted from nectin4, hSLAM, but not from dog SLAM.
The Stealth virus was then assayed for oncolytic activity against SK-OV-3 cells implanted in immunodeficient SCID mice. Some of the mice received passive immunization with human anti-MeV Abs. The Stealth virus reduced tumor growth more to higher extent than MeV. The anti-MeV inhibited the Mev-mediated reduction to higher extent than the Stealth-mediated reduction.
In the Discussion, the Authors foresee that in an immunocompent mouse model the T-cell response would much aid the antitumor efficacy of the recombinants. A conclusion I agree with.

The work is well conducted. The conclusions are supported by results. The paper is clearly written.

We would like to thank this reviewer for taking the time to carefully review our manuscript and for the positive assessment.

Reviewer #2: Considerable effort has been made in development of measles virus (MeV) as an oncolytic agent for treatment of cancer. However, pre-existing immunity in humans limits its efficacy. A previous report showed that substitution of the related canine distemper virus (CDV) envelope proteins avoided the effects of neutralizing antibodies. However, this virus has undesirable receptor specificities, which the present study addresses by engineering CDV H protein to be specific for CD46 (the receptor for attenuated strains of MeV, that is overexpressed on many cancers) and to lack binding to human Nectin-4. The approaches for re-targeting by fusing sFv fragments to the H protein and mutating its normal receptor binding sites have been developed previously. However, achieving the desired result would be a significant advance. The strength of the study consists of preliminary evidence for the desired specificity in the new virus (Fig. 4C) and its oncolytic activity in vivo in the presence of passive immunization with anti-MeV serum (Fig. 5 D or E [mislabeled]). The weaknesses of the study are that the manuscript is not clearly written and most of the data need major modifications.

We thank the reviewer for his/her critical analysis of our work and insightful questions. We have added new Figs 4B and 4C in which we generated additional Stealth viruses based on scFvs with lower binding affinity to CD46 (as determined by surface plasmon resonance) than those previously described. These additional Stealth viruses exhibited lower *in vitro* infectivity via CD46 receptors whereas cell entry via the pseudoreceptor HIS remained comparable, thus demonstrating cell target specificity of the viruses. We have also examined the role of receptor binding affinity, which can be used as a proxy for specificity, on the oncolytic activity of the Stealth virus. Our data, presented as new Fig. 5 show that the oncolytic activity of Stealth is driven by high affinity binding to CD46.

Reviewer #3: Steve Russel and colleagues present a nice study to show that a stealthed form of the measles OV MeV is capable of resisting neutralization by human serum antibody (passive immunization) while retaining the ability to slow the growth the growth of ovarian (orthotopic) and myeloma (xenograph) tumor models in mice. The “stealth” vector was produced by pseudotyping the MeV strain with glycoproteins (H/F) from a related but antigenically distinct virus, canine distemper virus (CDV). Vector retargeting was achieved by fusing CDV-H to a CD46 specific single chain antibody fragment and the F protein was modified to avoid recognition of nectin-4 and functioning well with the retargeted H glycoprotein. The tumor models expressed CD46. This is a good approach to avoiding initial virus inactivation during systemic delivery. An interesting finding was that the affinity for the targeted receptor played a role in fusogenic activity.

We thank the reviewer for this cogent summary.

Comments:
1. Can the stealth vector be used in repeat dosing experiments or does the animal create new neutralizing antibody. This is of interest since many vectors will require more than one administration. Could be done in immune competent mice rather than tumor models.

Indeed, MeV-Stealth induces neutralizing antibodies that do not cross-react with MeV. We have added a new Supplementary Fig8, in which HuCD46Ge-IFNAR^KO^ mice, deficient for type I IFN receptor and transgenically expressing human CD46, were inoculated intraperitoneally with either the vaccine lineage MeV or MeV-Stealth. The data showed that these mice generated non-cross-reactive neutralizing antibody responses against both viruses. Thus, MeV-Stealth virus would be more suitable for a one-shot therapy.

2. While the mouse system did not allow evaluaton of anti-tumor immunity, can this be done with humanized NSG mice and xenographs. The lack of replication in mice also makes it difficult to evaluate safety. Do the authors believe that the new vector envelope constructs might induce anti-tumor immunity comparable to the MeV strain. Since different surface glycoproteins are in play the nature of immune cell recruitment could be different as well as abscopal activity in an immune responsive animal system.

As we indicated in the discussion, one of the weaknesses of our study was our inability to examine the immunotherapeutic phase due to the lack of an informative immunocompetent rodent model. However, MeV-Stealth retains all the internal measles virus (MeV) replication machinery and structural genes since we only replaced the virus coat proteins F and H with the homologous F and H proteins from canine distemper virus (CDV). Interestingly, De Vries et al. have recently shown that MeV infection can lead to the induction of cross-genus-reactive T cells, which are particularly reactive with CDV (PMID 32636246). This finding, added to the fact that N protein is the main target of T-cell responses (PMID: [28904342](https://pubmed.ncbi.nlm.nih.gov/28904342/?utm_source=gquery&utm_medium=referral&utm_campaign=CitationSensor?otool=None)), led us to hypothesize that anti-tumor immunity would be comparable between the two viruses.

3. Is the stealth vector effective if targeted to nectin-4 that is likely to be present on many tumor types.

We appreciate this reviewer’s comment. Indeed, the usage of NECTIN4 over CD46 in oncolytic measles viruses is matter of debate in the oncolytic field. Work on non-small cell lung carcinoma indicated that CD46 governs oncolysis over NECTIN-4 (PMID: [25157763](https://www.ncbi.nlm.nih.gov/pubmed/25157763)) but we acknowledge this finding may not be applicable to other tumors. In the current work, we showed that CD46 tropism is sufficient to prolong survival in the ovarian cancer model we used (and myeloma cells do not express significant amounts of NECTIN4). This conclusion is based on the observation that Stealth showed oncolytic activity comparable to MeV, which can use NECTIN4 in addition to CD46. Thus, our data do not support a potential advance for the use of NECTIN-4 receptor over CD46. Importantly, elimination of NECTIN-4 tropism may have relevance to biosafety and the transmissibility of the virus. NECTIN4 is known to be the key receptor involved in MeV transmissibility and a Stealth virus with tropism for NECTIN4 could potentially be shed into the environment.

4. Since tumor killing is not complete, are resistant cell types selected that lack target receptor or is this a matter of insufficient replication and vector spread.

In order to maximize our chances to discern differences in the oncolytic activity of Stealth over MeV, we used much lower virus doses (~10 times lower) than were previously shown to be curative (PMID: 12183422). We therefore conclude that the incomplete killing in these studies is due to insufficient replication and vector spread. The selection or resistant cell types would not be apparent in the studies we have done to date.

5. IN Fig.4, why does the stealth virus replicate in dogSLAMF1 cells?

It is well established that the measles virus hemagglutinin can bind to SLAMF1 from human, cow, tamarin and dogs and that the virus exhibits similar entry activity in dog or human SLAM-expressing Vero cells (PMID: 31430904,11390585, 1200892). Indeed, some groups are using Vero cells expressing the dog SLAMF1 over human SLAMF1 (PMID: 23255805). Our results confirm this promiscuity of MeV for SLAMF1 from different species but also show that CDV does not utilize humanSLAMF1 to gain entry into cells, which is a potential safety advantage.

6. Is the stealth vector equally sensitive to innate tumor immune responses as MeV.

We believe the comment is duplicated and refer the reviewer to comment#2.

7. What role does ADCC play in curbing MeV activity-is it just VN.

Neutralizing antibodies detected by virus neutralization assays are considered the best correlate of protection against measles. This has been shown in epidemiological studies as well as in animal models. (PMID**:** [**24736226**](https://www.ncbi.nlm.nih.gov/pubmed/24736226); **2230231**). Another indication of the importance of neutralizing antibodies is the fact that seroconversion after vaccination is inhibited by maternal antibodies, which leaves children susceptible to infection (PMID: **909009); and that** passive transfer of neutralizing antibodies is protective both in humans exposed to the virus and in animal models (PMID: 16211552). We have updated the text to acknowledge our direct-virus neutralization centrist approach as follows:

“*A second weakness of the study relates to the mechanism of antibody mediated protection against measles. Here we focused exclusively on direct virus neutralization due to the good correlation between antibody titers, as determined by virus neutralization assay, and protection from measles (64-66). Thus, we did not evaluate alternative mechanisms of antibody mediated protection such as antibody-dependent complement-mediated cytolysis and antibody-dependent cellular-mediated cytotoxicity (ADCC)(67-69)*”

8. Is there a difference in stealth virus VN titers in the absence and presence of complement. What role does complement play in virus neutralization.

Virus neutralization titers, as determined on Vero cells, were not affected by the presence or the absence of the complement in contrast to our previous report on vesicular stomatitis virus (PMID: [24648451](https://www.ncbi.nlm.nih.gov/pubmed/24648451)). *In vivo* though, we believe that complement-mediated lysis (which may be increased following CD46 downregulation) has the potential to enhance virus oncolysis (PMID: [8523534](https://www.ncbi.nlm.nih.gov/pubmed/8523534)). Our inability to test this hypothesis in an immunocompetent mouse model is clearly a weakness of our study, as we indicated in the original discussion,

9. While the authors show that in fig 5 D, the stealth virus is resistant to VN there is a small difference in VN treatment and untreated although not significant. Wondering if some epitopes are shared with MeV that will block replication. Nevertheless Suppl fig 6 is convincing.

Since we used undiluted pooled human serum with high titers of neutralizing antibodies (3IU/mL), there might be some unspecific inhibition. However, we have repeated the experiment twice with similar results. We have added Fig. 7C and 7D from an additional experiment where we quantified tumor burden in the ovarian-tumor model after virus treatment in the presence or absence of anti-MeV antibodies. Statistical analysis for the bioluminescence quantification does not support inhibition of Stealth by anti-MeV antibodies. The raw numbers are provided as a source data file. In addition, and related to comment#1, we included as supplementary figure 8 cross-neutralization results of mouse antisera generated by infection with either MeV or Stealth. As we observed before with human or ferret antisera, we do not detect significant cross-reactivities in the mouse model.

**Part II – Major Issues: Key Experiments Required for Acceptance**
Please use this section to detail the key new experiments or modifications of existing experiments that should be absolutely required to validate study conclusions.

Generally, there should be no more than 3 such required experiments or major modifications for a "Major Revision" recommendation. If more than 3 experiments are necessary to validate the study conclusions, then you are encouraged to recommend "Reject".

Reviewer #1: 1. My major concern regards the genetic stability of nectin4- and SLAM- detargeting through single amino acids substitutions. How stable is the virus if serially passaged (let’s say 10 times) in human cancer cells simultaneously positive for CD446, nectin4 and SLAM?. Would some of the virus revert to nectin4 or SLAM usage?

Please be aware that Stealth was only detargeted from nectin4 because as we indicated in line 156, it was the only human receptor Stealth could use. Our tropism analysis in the context of the virus further confirmed that Stealth was unable to use human SLAMF1 (Fig.4F, see also PMID: **31430904).**

**In relation to the experiment suggested by the reviewer, and to the best of our knowledge,** a cell line expressing all three receptors does not exist. Even if we generate it in the lab, the results could be confounded due to differing expression levels of each receptor since binding avidity is directly correlated to receptor density. Stealth can naturally use dog SLAMF1 but not human SLAMF1 (Fig. 4F) and we assessed a potential adaptation of Stealth to use SLAMF1 in view of Bieringet et al.’s study (PMID: **23554862**). These authors reported adaptation of CDV to use human SLAMF1 within three passages and we therefore assessed whether the same was true for our virus. Our results, carried out using the method used by Bieringet et al., support a higher genetic stability of the virus or an intrinsic difference between CDV envelopes. As we indicated in the manuscript, the number of passages we used for the adaptation study (8) was shown to suffice to induce cyclical adaptation of measles virus quasiespecies (PMID: **30768648**). In a more recent research article. Leber et al, (PMID: **32053093) showed not differences in the accumulation of mutations of the vaccine-lineage measles virus on Vero cells versus a number of cancer cell lines. Of note, they only detected 19% of amino acid changes in the hemagglutinin versus 10% for the eGFR reporter gene (MeV relative gene sizes 12% and 5% respectively). Nevertheless,** as reviewer #3 indicated, a Stealth virus able to use NECTIN4 might prove beneficial for treatment of specific cancer types.

2. On a similar line of reasoning, to what extent is the Stealth virus really retargeted/detargeted? verification of tropism retargeting (fig. 4) should be performed also on human cells deficient for one or the other of the 3 receptors.

Chinese Hamster Ovarian cells (CHO) expressing individually each of the different receptors are used as the gold standard to determine virus tropism. To fulfill this reviewer’s request, we have included Figs 4B, 4C; Fig. 5 and Supplementary Figure 7., where we provide further evidence of the retargeting of Stealth both *in vitro* and *in vivo.* Stealth-N1E, encoding the scFv N1E with lower binding affinity to CD46 than A09, did not prolong survival in an ovarian cancer model. *In vitro*, Stealth-N1E infected CHO-CD46 with lower efficiency compared to Stealth-A09, whereas both viruses were able infect Vero-antiHIS cells similarly. We believe these results further emphasize the retargeting capabilities of the Stealth virus.

3. The Authors refer to avidity and affinity of the antibodies and/or retargeting moieties. None of the assay that the Authors applied (co-immunoprecipitation, ELISA) actually measures affinity of binding. The sentences should be rephrased, to avoid avidity, affinities, etc.

We agree with this reviewer’s comment that the ELISA does not provide reproducible binding affinity data to be compared across laboratories. For this reason, we are now including the binding affinity constants for the interactions of the scFvs with CD46 which were measured using surface plasmon resonance. The results corroborate our previous hypothesis based exclusively on the ELISA. In relation to the co-immunoprecipitation analysis, it does also semiquantitatively determine the interaction between two molecules. As an illustration, we refer this reviewer to two papers by Fag Li’s group where they achieved similar conclusion using BIAcore and co-immunoprecipitation analysis (PMID: [32225175](https://pubmed.ncbi.nlm.nih.gov/32225175/?utm_source=gquery&utm_medium=referral&utm_campaign=CitationSensor?otool=None), 32376634).

4. Fig. 4 B. the results are affected by the affinity of the antibodies being employed for detection. The Authors should in addition provide a PFU/genome copy number determination.

We appreciate the interesting suggestion. Even thought we did use different antibodies with the purpose of confirming virus identity, the same membrane was probed also using an antibody against MeV-N, which is shared between the viruses. Due to the fact that the N protein is the first transcriptional unit on the genome, its protein expression levels offers a good proxy for particle counts and therefore gives insight into any imbalance in the pfu/particle ratio while allowing us also to compare our results with those previously reported, (PMID: 26984727, 17804513 and 15685166)

*“We have now updated the text to emphasize this point: “Since we analyzed equivalent numbers of virus particles, our data suggest similar particle-to-plaque-forming unit (PFU) ratios for the two viruses, indicative of efficient incorporation into the virions of the foreign envelope”*

5. Fig. 4 A. There is some background virus growth in Vero-His. Can this virus be serially passaged? and what would be the receptor usage for the passaged virus: is the retargeting somehow leaky?

We believe this reviewer refers to background levels on Vero cells instead of Vero-antiHIS. Please note that the virus titers we reported do not even correspond to the virus input levels we originally used for the infection (4e5 cells x MOI 0.03= 1.2e4 FFU). Since there is not increase in virus titer over time, the low levels detected could probably be attributed to pseudo infection of remaining particles not removed after the absorption phase.

Although virus fusion with the host plasma membrane is the main mechanism governing entry of measles virus into susceptible cells, we cannot exclude the possibility of endocytic uptake of virus particles attached via weak cross-reactive binding to monkey CD46 (PMID 28100610 and 28250131).

In relation to the question as to whether the virus can be serially passaged, we believe the answer is implicit in the experiment summarized in Figure 4G, when we tried to adapt the virus to grow on Vero-hSLAMF1. In this case, we had to perform blind passages since eGFP-based fluorescence was barely detectable.

6. The Authors quote a very interesting paper from Zamarin group (Ref 55) which showed that some extent of prior immunity can be also beneficial. In this case, for example, what would be the effect of a prior T-cell immunity, as opposed to passive immunization with Antibodies? I would welcome a discussion in which the Authors address different facets of prior immunity, and the limits of passive immunization with Abs only.

- We appreciate this reviewer’s opinion on our discussion and we apologize for our rather succinct discussion on the subject for the sake of keeping the whole discussion unbiased. We indeed anticipate a synergistic effect of MeV-Stealth treatment in MeV-immunized subjects. Our hypothesis is based on the fact that 1) there are cross reactive T-cell epitopes between measles virus and canine distemper virus envelope glycoproteins (PMID [32636246](https://pubmed.ncbi.nlm.nih.gov/32636246/?utm_source=gquery&utm_medium=referral&utm_campaign=CitationSensor?otool=None)) and 2) T-cells responses do not prevent initial infection but do facilitate clearance measles virus. (PMID: **24736226)**

We have updated the discussion to reflect that among the measurable parameters of the immune response to MeV infection, we strictly focused on direct virus neutralization:

“*A second weakness of the study relates to the mechanism of antibody mediated protection against measles. Here we focused exclusively on direct virus neutralization due to the good correlation between antibody titers, as determined by virus neutralization assay, and protection from measles (64-66). Thus, we did not evaluate alternative mechanisms of antibody mediated protection such as antibody-dependent complement-mediated cytolysis and antibody-dependent cellular-mediated cytotoxicity (ADCC)(67-69)*”

Reviewer #2: 1. Most of the micrographs are not publication quality and are not readily interpretable in their current form. This includes Fig. 1A and B, Fig. 2A, Fig. 4C, and S5.

We apologize for the low quality of the figures due to size constrains upon submission. We are now uploading the figures individually at full-size resolution.

2. To the authors' credit, they do use one of (several) quantitative assays for cell-to-cell fusion mediated by cotransfection of H and F plasmids (Fig. 2B and D, Fig. 3B), although the basis for this one is not clearly described. However, statistical tests of one representative experiment performed in triplicate is not adequate to establish reproducibility. I know that normalization between luminescence experiments can be challenging, but typically involves comparison with a control.

We thank the reviewer for the opportunity to clarify this point. In our hands, the dual split-protein quantitative fusion assay, when compared with T7 promoter/ Luciferase assay, had better reproducibility and correlated the best with quantification of syncytium size. As this reviewer acknowledged, reproducibility does not mean 100% similar RU counts and we prefer to show raw rather than normalized data since statistical significance calculations based on low RU can be questionable. In light of this reviewer concern, we are providing a source data file. Furthermore, we are providing as supplementary figure 5B another biologic replicate for the quantitative fusion assay. As it can be observed, even though the RUs differ, the same pattern remained (the higher the binding affinity to CD46, the higher the fusion levels).

3. Flow cytometry data in Fig. S2 do not support the conclusion that the glycoproteins in transfected cells are expressed at similar levels. This requires analysis by mean fluorescence intensity, not percent transfected cells, which I presume to be represented (it is not stated). Also need repeats and statistics here as well.

- We apologize for inadvertently omitting the axis’ legend for the histograms. The histograms were included for direct comparison with the study from Bender et al., on the retargeting of the Nipah Virus envelope glycoproteins (PMID: **27281338). We have now updated the figure to include the mean fluorescence intensity and standard deviation of biological replicates rather than the values obtained for the specific histogram that we showed.**

4. Better quantification of the ability of the new recombinant virus to replicate in cells that express different receptors in Fig. 4 is required to conclude that "CD46-retargeted CDV envelope glycoproteins determine virus tropism."

A growth kinetic analysis of Stealth-A09 is provided in Figure 4D. Of note, even though Chinese Hamster Ovarian (CHO) cells can be made susceptible to measles virus infection, they are not permissive because of an intracellular block to later stages of the virus life cycle and we consider growth kinetic analysis futile on these cells.

To satisfy the reviewer concern, we rescued Stealth viruses expressing firefly luciferase instead of eGFP and provide luminescence quantification on cells infected with the viruses. Our data, summarized in Fig 4C, convincingly demonstrate CD46 targeting for the Stealth virus. In addition, we are including as Fig.5, a side by side comparison of the oncolytic activity of two Stealth viruses, which encoded either middle or high affinity CD46 binding scFv. The result in the ovarian cancer model indicates that the affinity of binding to CD46 drives virus oncolysis.

5. Fig. 5 does not present compelling evidence for the effectiveness of the new virus. Typically 5 mice are not sufficient to establish this. The luminescence images (Fig. 5D?) are not readily interpretable when presented like this.

We agree that we used a very low mice number. Our statistical significance is clearly determined by the magnitude of the response to the treatment and the effect size. The fact that we obtained a statistically significant prolonged survival with these low numbers strengthens the biological relevance of our findings. If we had needed a large animal number to achieve a statistical significance, the biological relevance of our results would be questionable. We are now providing a source data file for the reviewer to repeat the statistical analysis.

With relation to quantification of the bioluminescence images, quantification analysis was not performed since the signal was saturated due to the high tumor burden. We are now providing bioluminescence images from an additional experiment with their corresponding quantification and statistical analysis (Fig 5 and 7).

Reviewer #3: no specific experiments

**Part III – Minor Issues: Editorial and Data Presentation Modifications**
Please use this section for editorial suggestions as well as relatively minor modifications of existing data that would enhance clarity.

Reviewer #1: 7. Fig. 5. lettering of panels in text is confused. Please, correct
8. Line 778, please correct.

Thanks for pointing out the error.

Reviewer #2: 1. The Results section would benefit from a clearer presentation. I suggest the following for each experiment: 1) a clear statement of the purpose of the experiment, 2) enough explanation of the experimental approach for the reader to understand what was done and how the data are presented, 3) which results support the conclusion. In most cases, only the conclusion is stated.

We appreciate the reviewer’s comment towards improving the readability of our manuscript. We have revised this aspect of the paper to avoid detailed description of the results from control or supplementary figures – we hope this will help to avoid distracting readers from the most important result in each experiment.

2. A related problem is that the figure legends need to be sufficiently complete that the figure can be understood without reference to the text. This tends to overlap slightly with the second point above, and with the Materials and Methods section, but better that than the insufficient information provided.

We apologize for the figure legends not providing a complete and detailed description of how each experiment was performed, so that the reviewer had to read the Methods or Results to fully understand each experiment. We hope this reviewer finds the figure legends more descriptive now.

3. Minor edits are needed for correctness: cell-to-cell fusion is not the same as virus envelope fusion. This can be seen by the fact that even H/F combinations with little activity seem to function fine in the viruses from which they were derived. The cell-to-cell fusion assay is fine to test the effects of mutations, etc. Also, co-immunoprecipitation is not a measure of affinity or avidity.

Cell-cell fusion plays a major role in infectivity since the virus is strongly cell -associated. Indeed, we had considerable difficulty rescuing a Stealth virus with the homotypic H/F combinations from CDV. As we showed in Fig.2B, the heterotypic CD H/F combination was shown superior in terms of fusion activity and the virus encoding such a coat could be more easily rescued. As a matter of fact, lines 474-476 discussed the importance of fusogenicity in the generation of replication-competent MeV.

In relation to the use of protein pull-down to compare binding affinities, there is abundant literature reporting this assay with such a purpose. Corey E and Iorio R (PMID: 17626104) described the use of co-immunoprecipiation analysis to detect low-avidity associations between the envelope glycoproteins of paramyxoviruses. In the particular case of measles virus, Plemper et al. (PMID: [11967321](https://www.ncbi.nlm.nih.gov/pubmed/11967321)) used co-immunoprecipitation assays to determine that the strength of the envelope protein interaction modulates cell-cell fusion. Similarly, Bradel-Tretheway BG et al., (PMID: [30971473](https://pubmed.ncbi.nlm.nih.gov/30971473/?utm_source=gquery&utm_medium=referral&utm_campaign=CitationSensor?otool=None)) used the same conditions to demonstrate the previous conclusions applied also to Henipaviruses. More recently, the group of Fag Li has been able to determine using two different assays, surface plasmon resonance (PMID: [32225175](https://pubmed.ncbi.nlm.nih.gov/32225175/?utm_source=gquery&utm_medium=referral&utm_campaign=CitationSensor?otool=None)) and protein pull-down (PMID: 32376634), that the SARS-CoV-2 receptor binding domain (RBD) binds to hACE2 with higher affinity than SARS-CoV- RBD does, further validating this sort of approaches as a proxy of affinity measurement.

4. There are a number of misspellings and careless errors.

Our deepest apologies, we hope those misspellings and careless errors have been mitigated.

Reviewer #3: the review has a number of points that should be addressed by the authors.

Thanks for letting us know, we hope those points have been resolved.

PLOS authors have the option to publish the peer review history of their article ([what does this mean?](https://journals.plos.org/plospathogens/s/editorial-and-peer-review-process#loc-peer-review-history)). If published, this will include your full peer review and any attached files.


If you choose “no”, your identity will remain anonymous but your review may still be made public.


**Do you want your identity to be public for this peer review?** For information about this choice, including consent withdrawal, please see our [Privacy Policy](https://www.plos.org/privacy-policy).

Reviewer #1: **Yes:** Gabriella Campadelli-Fiume

Reviewer #2: No

Reviewer #3: No
