# Supplementary material for: MeV-Stealth: A CD46-specific oncolytic measles virus resistant to neutralization by measles-immune human serum
Source: PLoS Pathog. 2021 Feb 3;17(2):e1009283. doi: 10.1371/journal.ppat.1009283 (PMC7886131; doi:10.1371/journal.ppat.1009283)
Supplement: Attachment — Submitted filename: Response to reviewers II.docx [file ppat.1009283.s012.docx]

Please see below our point by point response to the reviewer comments on the revised version of our manuscript:

**Part I - Summary**
Please use this section to discuss strengths/weaknesses of study, novelty/significance, general execution and scholarship.

Reviewer #1: (No Response)

Reviewer #4: This is an interesting manuscript describing a novel clinically promising oncolytic MEV-CDV chimeric virus from a top group in the field of oncolytic virotherapy. It should be noted that multiple previous studies described various MEV-CDV chimeras. The current manuscript presents the following new data:
1. The novel engineered oncolytic MEV-CDV chimeric virus (“MeV-Stealth”) specifically targets CD46-expressing cells.
2. The MeV-Stealth virus has diminished undesirable tropism for human NECTIN-4 and hSLAMF1
3. The antitumor efficacy of MeV-Stealth in myeloma and ovarian tumor-bearing mice was similar to that of vaccine-lineage MeV.
4. Unlike MV, treatment of ovarian tumors with MeV-Stealth significantly increased overall survival (compared with treatment with vaccine-lineage MeV).

Than you. The assessment is accurate

Although the revised manuscript describes a lot of data and addresses many of the original critical points, there are still several important major deficiencies that I describe in Part II.

See below for our response to these points in Part II

Reviewer #5: In the manuscript, entitled “MeV-Stealth: A CD46-specific Oncolytic Measles Virus Resistant to Neutralization by Measles-Immune Human Serum”, Muñoz-Alía et. al. show that a measles virus variant, containing an envelope glycoprotein from a canine distemper virus further modified to retarget to CD46, is capable of evading detection by neutralizing antibodies, present in human serum. The authors selected their stealth combination (CDV-H 5804/F SPA) based on higher fusion ability and showed that scFv-CD46 addition indeed direct the virus towards CD46 and not NECTIN-4.

This is correct

**Part II – Major Issues: Key Experiments Required for Acceptance**
Please use this section to detail the key new experiments or modifications of existing experiments that should be absolutely required to validate study conclusions.

Generally, there should be no more than 3 such required experiments or major modifications for a "Major Revision" recommendation. If more than 3 experiments are necessary to validate the study conclusions, then you are encouraged to recommend "Reject".

Reviewer #1: The Authors have satisfactorily addressed all the issues and concerns raised by this reviewer.

Thank you for this positive feedback

Reviewer #4: Although the revised manuscript describes a lot of data and addresses many of the original critical points, there are still several important major deficiencies:

1. RETARGETTING. The manuscript presents a large amount of data demonstrating targeting of MeV-Stealth to CD-46-expressing cells and de-targeting from human NECTIN-4 and hSLAMF1. Unfortunately, practically all the data are from various CHO cell lines expressing (or not) these genes. In fact, the originally submitted manuscript was criticized for the lack of proof using various human cells lines expressing or not CD46, NECTIN-4 and hSLAMF1. The authors responded that “Chinese Hamster Ovarian cells (CHO) expressing individually each of the different receptors are used as the gold standard to determine virus tropism. To fulfill this reviewer’s request, we have included Figs 4B, 4C; Fig. 5 and Supplementary Figure 7…”. I do not agree with this response, and these figures do not show the requested data. CHO cells are very useful as a screening tool, but, ultimately, the novel tropism should be confirmed in a panel of non-malignant and malignant cell lines expressing one of these 3 proteins. Importantly, the expression levels and PTMs for these proteins could be very different in human and CHO cells, which could affect tropism in human vs CHO cells or human cancer vs human non-malignant cells. Interestingly, the same authors recently published a similar paper in Molecular Cancer Therapy describing CD38-targetted MEV-CDN chimeric virus (PMID: 32847970: “Retargeted and Stealth-Modified Oncolytic Measles Viruses for Systemic Cancer Therapy in Measles Immune Patients, Molecular Cancer Therapy 2020 Oct;19(10):2057-2067. doi: 10.1158/1535-7163.MCT-20-0134), in which they actually conducted this critical experiment (Fig. 3C: “Chimeric MV bearing CDV F and CDV H retargeted to EGFR or CD38 maintains receptor-specific infectivity in selected human tumor cell lines that express either EGFR or CD38.”)

We would like to thank this reviewer for taking the time to carefully review our manuscript. CD46 is expressed in all nucleated human cells and cell lines. Hence, the CD46-positive versus CD46-negative human cell comparison is not possible for this virus. The basis for CD46 receptor targeting is that CD46 receptor density is much higher on human tumor cells compared to non-tumor cells resulting in a much greater cell killing effect in tumor cells. This preferential infection and killing of cells with high CD46 receptor density is the basis for the tumor selectivity of Edmonston vaccine lineage measles viruses (PMID: 15256464; Anderson et al., 2004. Cancer Res. 64 (14): 4919-26). The goal of the specificity engineering in the current study was to generate an oncolytic measles virus with comparable CD46 tropism as for the parental MV-Edm but with the ability to evade serum neutralization by antimeasles antibodies in vaccinated individuals. Hence we needed to ablate all tropisms then build back in the CD46 specificity.

Nevertheless, to more fully address the concern of this reviewer regarding specificity on human cells we have added new supplementary figure 8 in which we infected a panel of immortalized and routinely used human cell lines with MeV-Stealth viruses displaying or not displaying a scFv against CD46. The new data demonstrates that productive infection of CD46-positive human cells is dependent on the display of an scFv with a ~20nM binding affinity to CD46, as we described in table 1. Secondly, we used the marmoset B-cell line B95 which are SLAMF1 positive but are known to lack the SCR1 domain of CD46 (which is targeted by the displayed anti-CD46 antibody). After we confirmed the receptor expression on this cell line, we showed B95 cells are not infected by MeV-Stealth but are infected by the parental MeV.

We have thus added the following text to the results (lines 203-208) as follows:

“We also observed an affinity-mediated CD46-dependent virus entry in immortalized cell lines routinely used in the laboratory. Again, virus infection in cells expressing human CD46 was observed with Stealth-A09 whereas that of an isogenic and untargeted MeV-Stealth (no scFv) or Stealth-K1/N1E was negligible. In B95m cells, a marmoset B-cell line widely used for the isolation of measles virus isolates, the lack of the SCR1 domain of CD46 greatly reduced Stealth-A09 infection. On the other hand, infection of B95m cells was readily observed with the unmodified/parental MeV.”

2. ONCOSELECTIVITY. Testing MeV-Stealth (and comparing it to the MV and at least some of the previously described MeV-CDN chimeras) in a panel of normal and malignant human cell lines is also important to demonstrate oncoselectivity of this novel virus MeV-Stealth. Due to the mentioned in the manuscript limitations of the in vivo murine system (MeV does not replicate in mouse cells), this critical question is not addressed in this manuscript.

The oncoselectivity of Edmonston lineage measles virus has been extensively studied and is in large part attributable to its CD46 receptor tropism. This was the rationale to build CD46 tropism back into the “stealthed” measles virus we generated in the current study. We have demonstrated that this engineered virus does indeed interact with the CD46 receptor, driving virus entry and subsequent intercellular fusion of infected with adjacent noninfected cells. It was therefore not considered necessary to repeat the exhaustive oncoselectivity studies that we previously conducted and published using CD46 tropic Edmonston strain oncolytic measles viruses (e.g., PMID: 15256464; Anderson et al., 2004. Cancer Res. 64 (14): 4919-26). The crtitical relationship between CD46 affinity and oncoselectivity was addressed *in vivo* on Figure 5, where we measured the oncolytic efficacy of Stealth viruses displaying CD46-specific scFvs with different affinities. We showed that only the virus displaying a scFv A09 achieved a clinical benefit as shown by a statistically significant enhanced mice survival (panel B) as well as reduced tumor growth (panel D). *In vivo* comparison between MeV and MeV-Stealth is shown in Figures 6 and 7. We further refer this reviewer to the above point#1 to address the comparison of different MeV-Stealth with MeV in different immortalized cell lines.

3. GENERAL EFFICACY IN VIVO. The demonstrated similar antitumor efficacy of MeV-Stealth to that of vaccine-lineage MeV is not so novel and was previously shown for other MeV-CDN chimeras.

We appreciate the comment but would like to remind the reviewer that the novelty and originality in this study was primarily to create a “stealthed” measles virus (i.e. resistant to neutralization by measles immune human serum) that retains the CD46 tropism known to be key to the oncoselectivity and oncolytic potency of MV-NIS, the oncolytic measles virus that has been most extensively tested in the clinic to date, which showed potent antitumor activity only in patients with unmeasurable antimeasles virus antibody titers. We do not believe we have failed to refer to key previous studies but would be happy to address this if the reviewer has specific suggestions.

5. EFFICACY IN VIVO IN THE RESENCE OF MEV AB. I am a little bit puzzled why MeV-Stealth antitumor efficacy was so clearly inhibited by MeV antibody (Fig. 7B)? Also, how did authors chose the dose of the ab? I noticed that the current manuscript used “600 mIU of anti-MeV IgG antibody intraperitoneally three hours before virus treatment”, while the mentioned above Molecular Cancer Therapy paper describing CD38-targetted MEV-CDN chimeric virus (PMID: 32847970) indicated “60 EU of pooled human measles-immune neutralizing serum added 3 hours before virus treatment”. Am I correct that 100 times less MeV ab was used in the current study? Why?

We believe the reviewer is mistaken. Figs.7B and 7D show no significant inhibition of MeV stealth antitumor activity. Has the reviewer analyzed the source data with a different statistical method that supports his/her assertion that the “MeV-Stealth antitumor efficacy was so clearly inhibited by MeV antibody (Fig. 7B)”?

To the second point, the dose of antibody used in the current study was not 100-fold lower than that used in the previous study. The confusion may have arisen because the reviewer is comparing two different measurement units (EU and mIU/mL) and considering them equal. In brief, the calibration of neutralization titer to mIU/mL that we used in this work is described in the materials and methods. On the other hand, EU, used in the related manuscript and previous work from our lab (PMID: 19934299), is based on a semi-quantitative indirect enzyme immunoassay performed by The Mayo Clinic Serology Clinical Laboratory. The EU assay measures MeV-specific IgG, regardless or the presence or absence of neutralizing antibodies. Since both our work and the related one co-submitted used 200ul of undiluted human sera, we actually used the exact same amount of anti-MeV-antibodies.

Reviewer #5: One weakness in the story (to my opinion) concerns the method used to indicate particle-to-pfu ratios of the MeV-Stealth virus compared to the original MeV (lines 193-195, and lines 279 -280 in discussion) and Figure 4E. Although it could well be that there is indeed no difference in the incorporation of the foreign envelop in the Stealth virus, a western blot is not the correct experiment to show that for the following reasons:
- The authors do not use purified viruses, since they use virus stocks, freed from cells by freeze-thawing. (Although in the material section this point is not directly clear, without looking up the reference mentioned) With a western blot, the authors cannot exclude that virus proteins produced by the cells are present in the cleared supernatant. Did does not necessarily mean that the proteins are incorporated in the virions. Furthermore, the amount of proteins in cell lysates can vary between different virus productions even when “equivalent numbers of particles” are used.
- Also, the authors state in line 195 that they observe “no significant difference” in expression levels of the major structural protein N. In fig 4E, however, there seems more N-protein present in the Stealth virus lane compared to the MeV lane. Without quantification it is very difficult to say something about significance (especially with only one western blot).
- For the stealth virus, the authors use an anti-His antibody and for the wt MeV a different antibody, recognizing MeV H-protein. It is well known that not all antibodies detect proteins with the same affinity on western blots. This makes a direct comparison very difficult.
If the authors want to make a conclusive point that the Stealth virus has a similar particle-to-PFU ratio they should show this with additional experiments and use more, (sucrose cushion) purified viruses or Electron Microscopy to quantify the number of particles in both (wtMeV and Stealth-Mev) supernatants with equal amounts of infectious virions. This would also give some information on the quality of the virions (presence of defective particles compared to intact virions).

We appreciate the thorough comment of this reviewer in this regard. We avoided the term “purified virus” since even a sucrose cushion would not eliminate cellular proteins from the preparation. To clarify this issue, we are now refering to the figure caption as “vero cell lysate”.

Far from making a conclusive point on this regard, the western blot was included to visually illustrate that we are using two viruses with two different envelope glycoproteins that enclose a similar replication machinery. We believe this was necessary instead of merely describing that we confirmed the virus identity by Sanger sequencing. Since it seems this point is a distraction to our main message in the paper we have modified the text to eliminate all reference to particle-to infectivity ratio. Thus, the text now reads as follows: (lines 210-211):

“The virus was further propagated to produce virus stock and subjected to Sanger sequencing and protein composition analysis to confirm its identity (Figure 4D)”

**Part III – Minor Issues: Editorial and Data Presentation Modifications**
Please use this section for editorial suggestions as well as relatively minor modifications of existing data that would enhance clarity.

Reviewer #1: (No Response)

Reviewer #4: (No Response)

Reviewer #5: 1. Line 128, “the affinity of CDV-F SPA for CDV-H 5804 was slightly lower than that of CDV-F SPA for CDV-H SPA (Figure 2C)” Based on figure 2C alone, without any quantification, it is difficult to see a difference in intensity between SPA(M437) and 5804. Could the authors re-phrase this sentence or delete it and leave the interpretation up to the readers.

The sentence has been deleted.

2. Line 169, “(Supplementary Figure 5)”, I think this should be changed to Supplementary Figure 6

Thanks for pointing this out. This has been changed to Supplementary Figure 6.

3. Line 188, “(Figure 4C, Supplementary Figure 6)” This should be changed to Supplementary Figure 7 (I think)

Correct. It has now been modified accordingly.

4. Line 243, “our results, presented in Supplementary Figure 7”. This should be changed to Supplementary Figure 8.

Since we included a new supplementary figure 8, we are now alluding to this as Supplementary Figure 9.

5. Materials and Methods, section “SDS-PAGE and immunoblotting”
Lines 453-54, “Band quantification was carried out using the KwikQuant Image Analyzer 1.4. (Cat. # D1016, Kindle Biosciences, LLC)” I could not find any quantification data in the manuscript, only speculations; see comment with line 128. If it is not used in the manuscript, please remove the sentence or provide the data.

We have now eliminated that sentence.

6. Figure 3B. It is difficult to distinguish the black lines from the red lines.

We apologize for the problem but since the fitting we used is quite acceptable, any other color combination would likely render similar issues.

7. Line 824, “Figure 4. CD46-retargeted CDV envelope glycoproteins determine virus tropism. (D) Schematic” D should be changed to A

We have corrected the typo. We have additionally switched panels D and E to allude to them in the order they are referenced in the main text.

8. Line 840 also Figure 4; panel G. “Representative microphotographs are shown after infection for three or six days”. Figure only shows pictures from Day 3, but not day 6, as is stated.

We apologize for the lack of clarification. We have now indicated that day 3 is shown for dog SLAMF1 since syncytia formation occurred rapidly. On the contrary, day 6 was used for Vero and Vero human SLAMF1. We additionally replaced the term “canine” for “dog” since panel F used the latter term. The text in the figure caption now reads as follows:

“Representative microphotographs are shown after infection for three (Vero dog SLAMF1) or six days (Vero and Vero human SLAMF1)”

9. Supplementary Figure 4, panel B. Typo: Volumen instead of Volume.

We apologize for the typo. It has been corrected

PLOS authors have the option to publish the peer review history of their article ([what does this mean?](https://journals.plos.org/plospathogens/s/editorial-and-peer-review-process#loc-peer-review-history)). If published, this will include your full peer review and any attached files.


If you choose “no”, your identity will remain anonymous but your review may still be made public.


**Do you want your identity to be public for this peer review?** For information about this choice, including consent withdrawal, please see our [Privacy Policy](https://www.plos.org/privacy-policy).

Reviewer #1: **Yes:** Gabriella Campadelli-Fiume

Reviewer #4: No

Reviewer #5: No
